# Role of Ferroptosis in Anticancer Drug Resistance in Head and Neck Squamous Cell Carcinoma: A Systematic Review of Preclinical Studies

**DOI:** 10.3390/ijms27219622

**Published:** 2026-10-27

**Authors:** Cintia Eliza Marques, Everton Freitas de Morais, Nicolly Valente Felipe-Moreira, Bruno Cesar da Costa, Lilianny Querino Rocha de Oliveira, Edgard Graner, Ahmed Al-Samadi, Ricardo D. Coletta

**Affiliations:** 1 Graduate Program in Oral Biology, School of Dentistry, University of Campinas, Piracicaba 13414-903, SP, Brazil; c207099@dac.unicamp.br (C.E.M.); evertonf@unicamp.br (E.F.d.M.); n256658@dac.unicamp.br (N.V.F.-M.); b213961@dac.unicamp.br (B.C.d.C.); l265902@dac.unicamp.br (L.Q.R.d.O.); 2 Department of Oral Diagnosis, School of Dentistry, University of Campinas, Piracicaba 13414-903, SP, Brazil; graner@unicamp.br; 3 Department of Oral and Maxillofacial Diseases, Clinicum, Faculty of Medicine, University of Helsinki, 00014 Helsinki, Finland; ahmed.al-samadi@helsinki.fi; 4 Institute of Dentistry, School of Medicine, Faculty of Health Sciences, University of Eastern Finland, 70210 Kuopio, Finland

**Keywords:** ferroptosis, head and neck squamous cell carcinoma, drug resistance, SLC7A11, GPX4, systematic review

## Abstract

Head and neck squamous cell carcinomas (HNSCC) frequently develop anticancer drug resistance, limiting treatment efficacy. Ferroptosis, an iron-dependent form of regulated cell death driven by phospholipid peroxidation, is dysregulated in drug-resistant HNSCC cells and may contribute to tumor survival. This systematic review synthesized preclinical evidence on the role of ferroptosis in HNSCC anticancer drug resistance. The review followed PRISMA 2020 guidelines and was registered in PROSPERO (CRD420261286567). PubMed/MEDLINE, Embase, Scopus, and ScienceDirect were searched without language or date restrictions, and preclinical studies evaluating ferroptosis in HNSCC drug resistance were included. Study selection, data extraction, and risk-of-bias assessment were independently performed by two reviewers. Out of 1870 records, 22 studies published between 2016 and 2026 met the inclusion criteria. Most used acquired cisplatin-resistant cell lines. Resistant cells consistently exhibited enhanced antioxidant defenses and reduced lipid peroxidation, primarily involving the SLC7A11/xCT–GSH–GPX4 axis and KEAP1–NRF2 signaling. Pharmacological or genetic inhibition of these defenses restored drug sensitivity in several models. Causal evidence was strongest in studies using ferroptosis-inhibitor rescue or in vivo validation, although methodological reporting was frequently insufficient. In conclusion, ferroptosis suppression contributes to HNSCC drug resistance, supporting ferroptosis induction as a potential resensitization strategy. Clinical translation requires validation in clinically relevant models.

## 1. Introduction

Head and neck squamous cell carcinomas (HNSCC) comprise a heterogeneous group of malignant neoplasms arising predominantly from the mucosal epithelium of the oral cavity, pharynx and larynx. They are among the most common malignancies worldwide and represent a major cause of cancer-related morbidity and mortality [1]. In 2024, cancers of the lip and oral cavity, pharynx (oropharynx, nasopharynx, and hypopharynx), and larynx together accounted for an estimated 965,000 new cases and 454,000 deaths worldwide. Driven by population growth and aging alone, the global cancer burden is projected to increase by 67% by 2050 [2]. Tobacco smoking and excessive alcohol consumption remain the major risk factors, acting synergistically to increase the risk of developing the disease [3]. Over the past two decades, persistent infection with high-risk human papillomavirus (HPV) has emerged as an independent etiological factor for oropharyngeal squamous cell carcinoma. HPV-positive tumors exhibit distinct molecular and clinical characteristics, frequently affecting younger individuals with limited tobacco exposure and generally showing a more favorable prognosis and better response to treatment than HPV-negative disease [4]. Although substantial advances have been achieved in therapeutic modalities, including surgery, radiotherapy, chemotherapy, targeted therapies, and immunotherapy, the selective elimination of malignant cells while preserving normal tissues remains one of the greatest challenges in cancer treatment [5]. Most conventional systemic therapies were developed to induce apoptosis; however, the ability of cancer cells to evade apoptotic cell death is one of the major mechanisms underlying tumor progression, therapeutic failure, and the development of drug resistance [6]. In HNSCC, platinum-based chemotherapeutic agents, particularly cisplatin (DDP), remain fundamental components of standard treatment protocols, exerting their antitumor activity mainly through DNA crosslinking, induction of genotoxic stress, and activation of apoptosis. However, approximately one-third of the patients experience therapeutic failure associated with DDP resistance, contributing to disease progression and reduced survival [7]. The mechanisms underlying anticancer drug resistance are highly complex and involve multiple processes, including increased drug efflux, enhanced DNA damage repair, and evasion of apoptosis [8,9,10]. These limitations have stimulated increasing interest in alternative forms of regulated cell death, particularly non-apoptotic pathways, as potential therapeutic strategies to overcome treatment resistance and improve clinical outcomes [11].

In this context, ferroptosis has emerged as a promising area of investigation. Ferroptosis, first identified and named by Brent Stockwell and colleagues in a series of studies published between 2003 and 2012, is a distinct form of regulated cell death characterized by molecular and morphological features that differ from those of apoptosis and autophagy-dependent cell death [12,13,14]. Unlike other forms of reactive oxygen species-induced cell death, ferroptosis is triggered by the iron-dependent peroxidation of phospholipids containing polyunsaturated fatty acids, leading to the disruption of plasma membrane integrity when cellular antioxidant systems fail to prevent lipid peroxide accumulation [14,15]. This process results primarily from the inability of the antioxidant defense system, particularly the glutathione (GSH)-glutathione peroxidase 4 (GPX4) axis, to detoxify phospholipid hydroperoxides enriched with polyunsaturated fatty acids [16,17]. Morphologically, ferroptosis is characterized by marked mitochondrial alterations, including mitochondrial shrinkage, reduced or absent cristae, and increased outer membrane density, without the nuclear fragmentation typically observed during apoptosis [18,19]. The identification of ferroptosis has expanded the prospects for the development of novel therapeutic strategies targeting the metabolic vulnerabilities of tumor cells, particularly those that have acquired resistance to conventional therapies [20].

Although emerging evidence suggests that ferroptosis modulation may influence tumor cell sensitivity to anticancer drug therapy and contribute to overcoming resistance [21], the underlying mechanisms in HNSCC remain fragmented and incompletely understood. Furthermore, to date, no systematic review has critically synthesized the available preclinical evidence regarding the role of ferroptosis in HNSCC drug resistance. Therefore, this systematic review aimed to comprehensively summarize and critically appraise the current evidence, providing an integrated understanding of the underlying molecular mechanisms and the therapeutic potential of ferroptosis modulation as a strategy to overcome resistance.

## 2. Methods

### 2.1. Protocol and Registration

This systematic review was conducted according to the Preferred Reporting Items for Systematic Reviews and Meta-Analyses (PRISMA 2020) guidelines [22]. The review protocol was prospectively registered in the International Prospective Register of Systematic Reviews (PROSPERO), and received registration number CRD420261286567.

### 2.2. Review Questions and Study Framework

The review aimed to answer the following questions: (1) What is the role of ferroptosis in anticancer drug resistance in HNSCC?; (2) Which molecular mechanisms regulate ferroptosis in resistant HNSCC models?; and (3) Can modulation of ferroptosis restore sensitivity to conventional anticancer drug therapy in preclinical HNSCC models? To address these questions, the review was structured according to the PICO framework, as follows: Population: preclinical in vitro and in vivo models of HNSCC; Intervention: experimental modulation of ferroptosis and/or treatment with ferroptosis-inducing agents in the context of drug resistance; Comparison: untreated controls, therapy-sensitive models, or alternative experimental conditions; Outcomes: evidence of ferroptosis activation or inhibition, mechanisms underlying resistance, and changes in therapeutic response.

### 2.3. Search Strategy

A comprehensive literature search was performed in the electronic databases PubMed/MEDLINE, Embase, Scopus and Science direct. In addition, the reference lists of all included studies and relevant reviews were manually screened to identify potentially eligible articles. The search strategy combined controlled vocabulary (MeSH and Emtree terms) and free-text keywords related to head and neck squamous cell carcinoma and its anatomical subsites, ferroptosis, and resistance. No restrictions on language or year of publication were applied. The complete search strategy for each database is provided in Supplementary Table S1. Initially, the search included all articles published up to 28 February 2026. A second literature search using the same search terms was performed on 31 August 2026. Although additional records were identified, none met the predefined eligibility criteria and, therefore, no additional studies were included in the review.

### 2.4. Study Selection and Eligibility Criteria

Study selection was conducted independently by two reviewers using a two-stage screening process. First, titles and abstracts were screened according to the predefined eligibility criteria. Studies considered potentially eligible proceeded to full-text assessment. Disagreements between reviewers were resolved through discussion and, when necessary, consultation with a third reviewer. Studies were included if they investigated ferroptosis in the context of drug resistance using preclinical HNSCC models, including established or experimentally induced resistant cell lines and/or animal models. The following studies were excluded: (i) studies that did not evaluate drug resistance; (ii) studies that did not experimentally assess ferroptosis or its molecular regulators; (iii) studies using non-HNSCC cancer models; (iv) clinical studies, case reports, reviews, conference abstracts, editorials, and letters; and (v) studies based exclusively on bioinformatics or in silico analyses without experimental validation.

**Figure 1:**
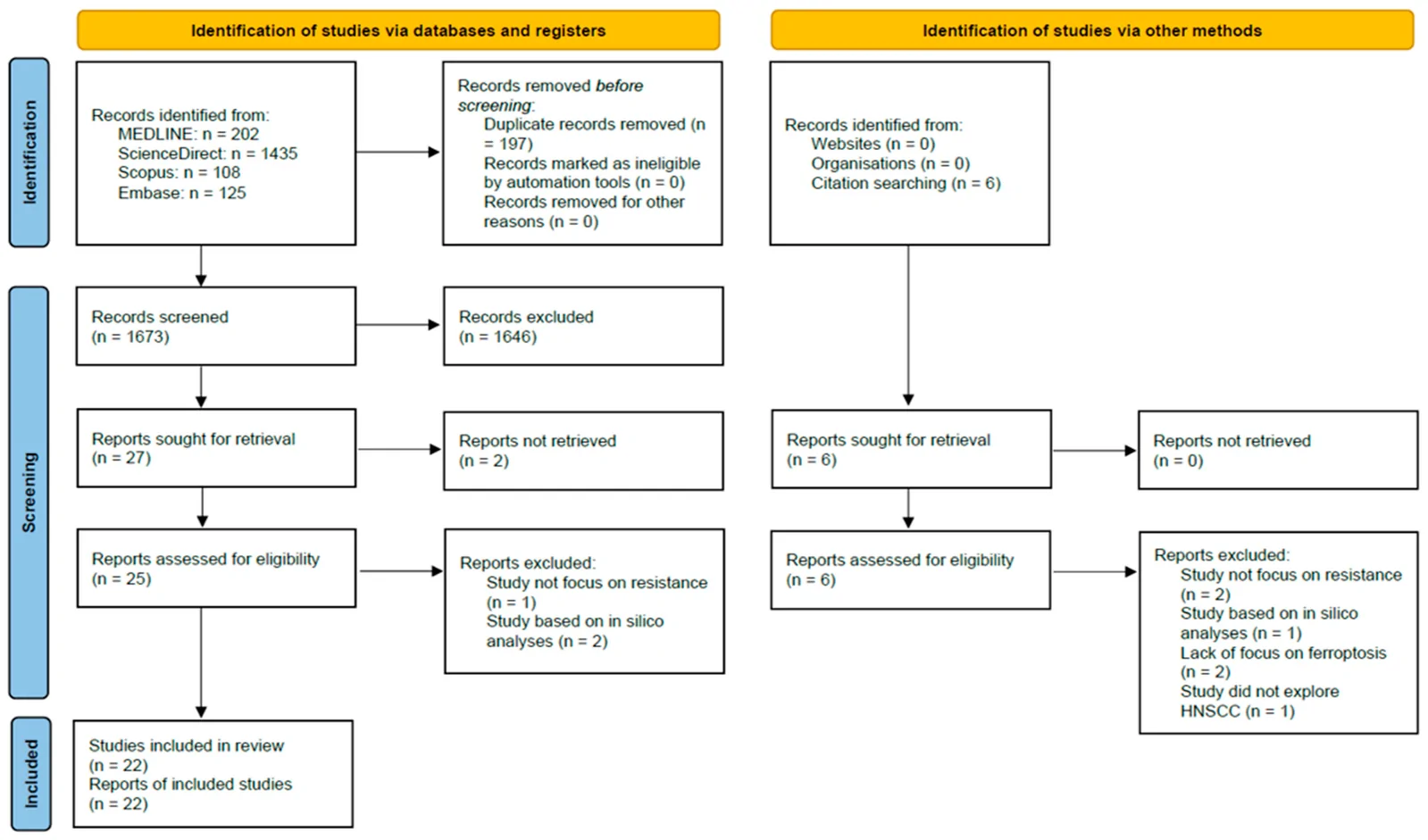
PRISMA flow diagram of literature search and selection criteria applied in this study.

The study selection process is summarized in the PRISMA 2020 flow diagram (Figure 1). A total of 1870 records were identified through the database searches. After duplicate removal, 1673 records remained for title and abstract screening, of which 25 were selected for full-text assessment. Other 6 articles, identified in the reference list of the selected articles, were fully assessed, but none met the inclusion criteria established for this review. Following eligibility evaluation, 22 studies met the inclusion criteria and were included in the qualitative synthesis [23,24,25,26,27,28,29,30,31,32,33,34,35,36,37,38,39,40,41,42,43,44]. The reasons for exclusion after full-text assessment are presented in Supplementary Table S2.

### 2.5. Data Extraction, Synthesis, and Risk of Bias Assessment

Data were independently extracted by two reviewers using a standardized data extraction form. The following information was collected from each included study: first author, year of publication, country, experimental model (in vitro and/or in vivo), characteristics of the cell lines or animal models, therapeutic agent, ferroptosis-related intervention, molecular targets and signaling pathways investigated, experimental outcomes, and the main findings. The experimental evidence supporting ferroptosis was classified as ferroptosis-associated cell death or markers only, based on the extent of functional and mechanistic evidence reported in each study. Ferroptosis-associated cell death was defined as reduced cell viability or increased cell death accompanied by direct evidence of lipid peroxidation and rescue by a specific ferroptosis inhibitor or iron chelator. In contrast, the markers-only category included studies reporting alterations in ferroptosis-related markers without direct evidence of lipid peroxidation, even when accompanied by functional evidence of cell death. The extracted data were subsequently summarized, categorized, and synthesized according to the objectives of the review.

For in vitro studies, methodological quality was independently appraised by two reviewers using an adapted GRADE approach [45]. Because no validated tool is currently available for appraising the certainty of evidence from in vitro studies, the GRADE framework was adapted to the individual study level to provide a structured appraisal of the methodological robustness of each experiment. Domains originally designed for the body of evidence were interpreted at the study level: inconsistency as internal consistency across experimental replicates and assays, and publication bias as selective reporting of results. Each domain was rated as “no serious limitations”, “serious limitations”, or “unclear” when the available information was insufficient for judgment, and the overall certainty of evidence for each study was classified on a four-level scale (very low, low, moderate, or high). For in vivo studies, methodological quality and risk of bias were independently assessed by two reviewers using the SYRCLE’s Risk of Bias tool [46]. Any disagreements were resolved through discussion or, when necessary, consultation with a third reviewer. Owing to the expected heterogeneity in experimental models, interventions, and outcome measures, the findings were synthesized qualitatively.

## 3. Result

### 3.1. Study Selection

The included studies were published between 2016 and 2026. Most were conducted in China, with additional contributions from France, Taiwan, the Republic of Korea, and the United States. Experimental approaches varied with respect to the tumor models, therapeutic agents, ferroptosis-modulating strategies, and methods used to assess ferroptosis. Most studies were performed using in vitro models, whereas a smaller number included validation in xenograft or patient-derived animal models. The main characteristics and principal findings of the included in vitro studies are summarized in Table 1, whereas the characteristics of the in vivo studies are depicted in Table 2.

Among the 22 included articles, 18 provided functional and mechanistic evidence supporting the involvement of ferroptosis suppression in anticancer drug resistance, whereas the remaining four reported changes in ferroptosis-associated markers in resistant cells without sufficient evidence to establish ferroptotic cell death. This classification was based on the presence of reduced cell viability/increased cell death accompanied by direct evidence of lipid peroxidation and rescue by a specific ferroptosis inhibitor or iron chelator. Studies reporting alterations in ferroptosis-associated markers without direct evidence of lipid peroxidation were classified as markers only.

### 3.2. Experimental Landscape of Ferroptosis in HNSCC Drug Resistance

Experimental studies investigating ferroptosis in HNSCC resistance have relied predominantly on models of acquired DDP resistance generated by continuous drug exposure. Following the pioneering work of Roh et al. [23], resistant derivatives have been established from HN3, HN4, HN9, CAL27, SCC9, HN30, HSC3, SCC15, and other HNSCC cell lines, although the protocols used to generate resistance differed considerably among studies [25,30,31,32,42]. Investigations involving resistance to other therapies, including cetuximab, gefitinib, methotrexate, and doxorubicin, were comparatively scarce [26,29,33,40,44]. A few studies also explored constitutive differences in ferroptosis susceptibility among HNSCC subtypes or evaluated drug-tolerant persister cells rather than experimentally generated resistant populations [23,33].

Methodological approaches evolved substantially over time. Early studies relied mainly on cell viability assays, glutathione depletion, ROS generation, and expression analysis of ferroptosis-related genes, whereas later investigations incorporated lipid-peroxidation probes, intracellular iron quantification, transmission electron microscopy, clonogenic assays, migration and invasion assays, pharmacological rescue experiments, and genetic manipulation. More recent studies further integrated bulk and single-cell transcriptomics, chromatin-accessibility profiling, whole-exome sequencing, and bioinformatic analyses of patient datasets [31,36,42]. Similarly, experimental models progressively gained translational relevance. Most in vivo studies used subcutaneous xenografts established from resistant cell lines, whereas orthotopic xenografts, patient-derived xenografts, and patient-derived organoids were introduced only recently [31,33,36]. Nevertheless, these advanced models remain underrepresented, and most mechanistic investigations continue to rely on conventional two-dimensional cell cultures.

Overall, the available literature is characterized by a predominance of DDP-resistant in vitro models followed by validation in subcutaneous xenografts. Although increasing methodological sophistication has improved the translational relevance of the field, orthotopic models, patient-derived systems, and comprehensive in vivo validation remain relatively uncommon.

### 3.3. Ferroptosis Regulatory Mechanisms in HNSCC Drug Resistance

The xCT–GSH system was the first ferroptosis-related mechanism linked to drug resistance in HNSCC. In HN3, HN4 and HN9 DDP-resistant cells, cystine deprivation or pharmacological inhibition of xCT with erastin and sulfasalazine depleted intracellular GSH, increased lipid peroxidation, and reduced cell viability [23]. Ferrostatin-1 (Fer-1), but not necrostatin-1, prevented these effects, and SLC7A11 silencing increased the response of resistant cells to DDP, while *N*-acetylcysteine attenuated the activity of the sulfasalazine–DDP combination. The same association was observed in vivo, where SLC7A11 knockdown enhanced DDP-mediated inhibition of resistant xenografts [23]. In a subsequent study, the same group demonstrated that the regulation of this antioxidant system involved the KEAP1–NRF2 pathway [24]. DDP-resistant HN cells expressed higher basal levels of NRF2 than their parental counterparts, and exposure to artesunate increased nuclear NRF2 and activated ARE-dependent targets, including HO-1 and NQO1. KEAP1 silencing reduced artesunate sensitivity, whereas NRF2 knockdown or trigonelline lowered GSH, increased ROS, and restored cell death by ferroptosis [24]. In CAL27-DDP and SCC9-DDP resistant cells, resistance was likewise accompanied by increased activation of NRF2, HO-1, and xCT, together with higher GSH levels and lower ROS accumulation and lipid peroxidation. Carnosic acid inhibited this signaling and increased DDP sensitivity; liproxstatin-1 or forced NRF2 expression attenuated both ferroptosis and chemosensitization [25]. A more complex form of NRF2 dysregulation was reported in HN30-R8 cells [31]. Osman et al. [31] identified acquired KEAP1 mutations and epigenetic activation of NRF2-regulated genes during the development of DDP resistance. NRF2 knockdown or restoration of wild-type KEAP1 reduced GPX4 expression, increased lipid peroxidation, and rendered resistant cells responsive to DDP- and RSL3-induced ferroptosis. Single-cell transcriptomic and chromatin-accessibility analyses showed that KEAP1–NRF2 pathway activation was restricted to a subpopulation of resistant cells rather than being uniform across the culture [31].

Iron storage and availability also influenced ferroptosis sensitivity. Liu et al. [26] found marked differences among HNSCC cell lines, with CAL33 and the laryngeal cell lines AMC-HN-8 and TU686 responding to erastin and RSL3, whereas the nasopharyngeal cell lines CNE-2, S18, and S26 were poorly responsive. FTH1 expression was higher in the resistant nasopharyngeal models, and FTH1 knockdown increased RSL3-induced cell death, including in CNE-2 cells. RSL3 also enhanced the effect of cetuximab in CNE-2 cells, which were resistant to both EGFR inhibition and ferroptosis induction [26]. The findings linked ferritin-mediated iron sequestration with intrinsic ferroptosis resistance, although the pattern of sensitivity varied with tumor site and RAS-family gene expression. OLFM4 was identified through transcriptomic comparison of parental and DDP-resistant patient-derived HNSCC organoids. In CAL27 and 5-8F cells, OLFM4 overexpression reduced DDP-induced ROS production, MDA accumulation, and Fe^2+^ levels and increased mitochondrial membrane potential, whereas OLFM4 knockdown produced the opposite changes. OLFM4 overexpression was also associated with increased LCN2, FTH1, SLC7A11, and GPX4 and reduced acyl-CoA synthetase long-chain family member 4 (ACSL4) expression, suggesting that OLFM4 suppresses ferroptosis through modulating iron homeostasis and reinforcement of the GSH-dependent antioxidant system, while also preserving mitochondrial function [36]. However, direct physical interactions between OLFM4 and the proposed ferroptosis regulators were not investigated, and the functional experiments were conducted mainly in conventional cell lines rather than in the resistant organoids used for target discovery. Moreover, ferroptosis suppression is not restricted to the SLC7A11–GPX4 pathway [34]. Drug-tolerant persister cells derived from HSC3 and HSC4 cells exhibited higher levels of ferroptosis suppressor protein 1 (FSP1). FSP1 and lipid metabolism-related genes, and FSP1 silencing reduced stemness, invasion, and metastatic traits, while increasing ferroptosis susceptibility and enhancing the effect of DDP in a patient-derived xenograft model [34].

Post-translational regulation provided another link between ferroptosis and resistance. USP14 stabilized FABP5 by preventing its proteasomal degradation, thereby suppressing ferroptosis, whereas USP14 inhibition enhanced DDP response and tumor suppression in xenografts [37]. In another study, Ma et al. [30] identified circ_0000140 as an upstream regulator of SLC7A11 in DDP-resistant OSCC. Upregulated circ_0000140 promoted DDP resistance by sponging miR-527 and sustaining SLC7A11 expression, thereby suppressing ferroptosis. Zhang et al. [43] identified the miR-214-3p/GPX4 axis as a regulator of DDP resistance in OSCC. miR-214-3p restoration suppressed GPX4, increased ROS and Fe^2+^ levels, reduced DDP IC_50_, and inhibited xenograft growth, whereas GPX4 overexpression reversed these effects. Epigenetic control of lipid peroxidation was described in CAL27/DDP cells [42]. The authors demonstrated that sodium butyrate restored DDP sensitivity by inducing ferroptosis, increasing ROS, lipid peroxidation, Fe^2+^ accumulation, and ferroptotic mitochondrial changes.

Taken together, the studies identified the SLC7A11–GSH–GPX4 axis as a central regulator of ferroptosis resistance in HNSCC. Additional mechanisms, including NRF2 activation, FTH1-mediated iron sequestration, FSP1-associated persistence, non-coding RNA regulation, and HDAC9–EGR1–POR signaling, contributed to ferroptosis suppression and DDP resistance. Although most pathways were supported by genetic or pharmacological validation, some mechanisms, particularly OLFM4-associated regulation, require further confirmation.

### 3.4. Therapeutic Modulation of Ferroptosis in HNSCC Drug Resistance

Therapeutic strategies targeting ferroptosis in HNSCC resistance were primarily aimed at disrupting antioxidant defenses. Roh et al. [23] showed that SLC7A11 silencing, erastin or sulfasalazine increased lipid ROS, depleted intracellular GSH, and restored DDP sensitivity in HN3-cisR, HN4-cisR, and HN9-cisR cells, while enhancing tumor suppression in resistant xenografts. Fer-1, but not necrostatin-1, prevented these effects, confirming ferroptosis as the predominant mechanism. More recently, Liu et al. [32] identified the TCF12–OTUB1–SLC7A11 axis as another mechanism regulating DDP response. TCF12 restoration promoted SLC7A11 ubiquitination, enhanced ferroptosis, reduced the DDP IC_50_, and suppressed the growth of resistant xenografts, whereas SLC7A11 overexpression reversed these effects.

NRF2-mediated antioxidant adaptation was subsequently targeted using artesunate, carnosic acid, and genetic approaches. Artesunate induced ferroptosis but showed reduced activity in NRF2-active resistant cells, whereas NRF2 direct silencing restored sensitivity in vitro and in vivo [24]. Similarly, carnosic acid sensitized CAL27-DDP and SCC9-DDP cells to DDP by inhibiting the NRF2/HO-1/xCT axis, although evidence remained limited to in vitro models [25]. In HN30-R8 cells, NRF2 inhibition, KEAP1 restoration, or GLS1 blockade disrupted antioxidant capacity, restored DDP sensitivity, and reduced metastatic progression in an orthotopic model [31].

Alternative ferroptosis defense mechanisms were targeted through FSP1 and USP14 inhibition. iFSP1 increased ferroptotic susceptibility of DDP-tolerant persister cells, reduced stemness- and invasion-related phenotypes, and improved DDP efficacy in a patient-derived xenograft model, supporting FSP1 as a mediator of treatment persistence [34]. USP14 inhibition destabilized FABP5, increased ferroptosis markers, suppressed proliferation, migration, and invasion, and enhanced DDP-mediated tumor suppression in CAL27 xenografts [37]. Epigenetic and RNA-based strategies provided additional therapeutic approaches. Sodium butyrate restored DDP sensitivity in CAL27/DDP cells through HDAC9 inhibition and activation of the EGR1–POR axis, while Fer-1 rescue confirmed ferroptosis-dependent chemosensitization both in vitro and in xenografts [42]. Genetic modulation of non-coding RNAs also restored ferroptotic susceptibility: circ_0000140 promoted DDP resistance through the miR-527/SLC7A11 axis, whereas miR-214-3p restoration suppressed GPX4 and increased DDP response in OSCC models [30,43]. Nanotechnology-based approaches combined ferroptosis induction with conventional therapies. DMEFe nanoparticles enhanced doxorubicin activity by increasing ROS and suppressing NRF2/SLC7A11/GPX4 signaling, although they were not tested in resistant models [33]. Similarly, Sor-Ce6 nanoparticles, in which sorafenib acted as a ferroptosis inducer, depleted GSH and downregulated GPX4 in CAL27 cells [35].

Overall, therapeutic interventions ranged from direct inhibition of the cystine/glutamate antiporter to modulation of NRF2 signaling, glutamine and lipid metabolism, protein stability, epigenetic regulation, non-coding RNAs, and nanoparticle-based delivery systems. Across the included studies, ferroptosis modulation consistently increased DDP sensitivity and, in several models, also reduced proliferation, migration, invasion, stemness-related features, tumor growth, and metastatic dissemination. The strongest evidence involved disruption of antioxidant defenses through xCT, NRF2, GLS1, FSP1, USP14, and HDAC9-associated pathways and was supported by resistant models, ferroptosis-rescue experiments, and in vivo validation. Nevertheless, most interventions remain preclinical, and the limited use of orthotopic models, patient-derived systems, RNA delivery platforms, and in vivo ferroptosis validation remains a major barrier to clinical translation.

**Table 1:** Main characteristics of the included in vitro studies investigating ferroptosis and resistance in head and neck squamous cell carcinoma.

Study (Year)	Cell Lines	Therapeutic Context	Resistance Model	Ferroptosis Modulation	Main Pathway	Evidence of Ferroptotic Cell Death	Main Findings
Roh et al. [23]	AMC-HN3, HN4, HN9; HN3-cisR, HN4-cisR, HN9-cisR	Cisplatin	Acquired cisplatin resistance	Erastin, sulfasalazine, SLC7A11 silencing	xCT/SLC7A11–GSH	Ferroptosis-associated cell death	xCT inhibition induced ferroptosis and sensitized cisplatin-resistant cells to cisplatin
Roh et al. [24]	HN2–HN10, SNU cell lines; HN3, HN4, HN9; HN3-cisR, HN4-cisR, HN9-cisR	Artesunate; cisplatin used to generate resistant cells	Acquired cisplatin resistance	Artesunate, trigonelline, NRF2/HO-1/KEAP1 silencing	KEAP1–NRF2–ARE	Ferroptosis-associated cell death	NRF2 activation reduced artesunate-induced ferroptosis, whereas NRF2 inhibition restored sensitivity in cisplatin-resistant cells
Han et al. [25]	CAL27, SCC9, CAL27/DDP, SCC9/DDP, NHOK	Cisplatin	Acquired cisplatin resistance	Carnosic acid, liproxstatin-1, NRF2 overexpression	NRF2/HO-1/xCT	Ferroptosis-associated cell death	Carnosic acid induced ferroptosis and restored cisplatin sensitivity by inhibiting NRF2/HO-1/xCT signaling
Liu et al. [26]	CAL33, AMC-HN-8, TU686, CNE-2, S18, S26; N2A and NSCs	Cetuximab	Ferroptosis and EGFR-inhibition resistance	RSL3, erastin, sorafenib, liproxstatin-1, FTH1 silencing	FTH1/iron homeostasis	Ferroptosis-associated cell death	FTH1 contributed to ferroptosis resistance, and RSL3 sensitized cetuximab-resistant cells to cetuximab
Wang et al. [27]	Hep2, Detroit562, TU686; NP69-SV40T	Gefitinib	Gefitinib resistance	RSL3, erastin, EMP1 modulation	EMP1–Hippo/YAP1–Rac1–NOX1	Ferroptosis-associated cell death	EMP1 enhanced ferroptosis and increased gefitinib sensitivity through ROS and lipid-peroxidation signaling
Zhou et al. [28]	CAL27, CAR, SCC15, HOK	Cisplatin	CAL27-derived cisplatin-resistant CAR cells	AEBP1 silencing, sulfasalazine	AEBP1/MAPK–SLC7A11/GPX4	Ferroptosis-associated cell death	AEBP1 knockdown increased ferroptosis and sensitized resistant cells to sulfasalazine and cisplatin
Jehl et al. [29]	CAL27, CAL33, SCC9; patient-derived HNSCC tumoroids T1 and T2	Cetuximab	No established resistant cell line	RSL3, metformin, EREG silencing	EREG–GPX4/c-Myc	Ferroptosis-associated cell death	EREG silencing and ferroptosis induction enhanced cetuximab activity in cell lines and tumoroids
Ma et al. [30]	SCC-15, CAL-27, SCC-15/DDP, CAL-27/DDP, HOK	Cisplatin	Acquired cisplatin resistance	circ_0000140 silencing, miR-527 modulation, erastin	circ_0000140/miR-527/SLC7A11	Markers only	circ_0000140 promoted cisplatin resistance by increasing SLC7A11 and suppressing ferroptosis
Osman et al. [31]	HN30-P, HN30-R8	Cisplatin	Acquired cisplatin and ferroptosis resistance	RSL3, ferrostatin-1, NRF2 silencing, KEAP1 restoration, IACS-6274	KEAP1–NRF2/GLS1	Ferroptosis-associated cell death	NRF2 activation suppressed ferroptosis and promoted cisplatin resistance; NRF2 or GLS1 targeting restored sensitivity
Liu et al. [32]	TSCCA, TSCCA/DDP, SCC-9, SCC-9/DDP, Tca8113, CAL-27; NHOK and HEK293T	Cisplatin	Acquired cisplatin resistance	TCF12 overexpression, RSL3, liproxstatin-1	TCF12–OTUB1–SLC7A11	Ferroptosis-associated cell death	TCF12 promoted ferroptosis by repressing OTUB1-dependent SLC7A11 stabilization and restored cisplatin sensitivity
Wang et al. [33]	SCC-25	Doxorubicin	No established resistant model	DOX-loaded EGCG–Fe nanoparticles	NRF2/SLC7A11/GPX4	Markers only	Nanoparticles induced ROS accumulation and ferroptosis, enhancing doxorubicin cytotoxicity
Wu et al. [34]	HSC3, HSC4; HSC3-P and HSC4-P drug-tolerant persister cells	Cisplatin	Cisplatin-tolerant persister state	iFSP1, FSP1 silencing	FSP1–ACSL4	Markers only	FSP1 inhibition reduced persister-cell traits and increased cisplatin-related vulnerability to ferroptosis
Xu et al. [35]	CAL-27	Sorafenib and photodynamic therapy	Hypoxia-related photodynamic therapy resistance	Sorafenib–Ce6–Fe nanoparticles	Fe^2+^/Fenton reaction–GPX4	Markers only	Nanoparticles induced ferroptosis and improved photodynamic efficacy under hypoxic conditions
He et al. [36]	5-8F, CAL27, SAS, SCC-9, NOKs; HNSCC-PDOs and HNSCC-PDOcis-R	Cisplatin	Cisplatin-resistant patient-derived organoids	OLFM4 overexpression and silencing	OLFM4–SLC7A11/GPX4	Ferroptosis-associated cell death	OLFM4 suppressed oxidative stress and ferroptosis, whereas its silencing restored cisplatin sensitivity
Qian et al. [37]	CAL27, TU686, FaDu, FaDu/DDP, TU212, SCC25	Cisplatin	Acquired cisplatin resistance	USP14 silencing, IU1, USP14 overexpression	USP14–FABP5–SLC7A11/GPX4	Ferroptosis-associated cell death	USP14 stabilized FABP5, suppressed ferroptosis and promoted cisplatin resistance
Wang et al. [38]	CAL27, CAR, SCC15, SCC9, HOK	Cisplatin	CAL27-derived cisplatin-resistant CAR cells	TPI1 silencing or overexpression, erastin, ferrostatin-1	TPI1–SLC7A11/GPX4/TFRC	Ferroptosis-associated cell death	TPI1 knockdown induced ferroptosis and reduced proliferation, invasion, EMT and cisplatin resistance
Wang et al. [39]	Cal27, HSC3	Cisplatin	No resistant cell line reported	CAY-10566	mTOR/SREBP1/SCD1–HMOX1	Ferroptosis-associated cell death	SCD1 inhibition induced ferroptosis and enhanced cisplatin sensitivity
Yu et al. [40]	A253, MOC1	Methotrexate	Resistance not evaluated	Methotrexate, ferrostatin-1	ERK–KEAP1–NRF2	Ferroptosis-associated cell death	Methotrexate induced ferroptosis through ERK-mediated KEAP1 activation and NRF2 degradation
Zhang et al. [41]	CAL-27, SCC-9; human oral keratinocytes	Cisplatin	Reduced cisplatin sensitivity; no resistant derivative	CAV1 silencing	CAV1–GPX4/FTH1/HO-1	Ferroptosis-associated cell death	CAV1 knockdown enhanced cisplatin-induced ferroptosis and reduced proliferation, migration and invasion
Wang et al. [42]	CAL27, CAL27/CDDP, CAL33, HSC3, SCC15; HEK-293	Cisplatin	Acquired cisplatin resistance	Sodium butyrate, erastin, ferrostatin-1	HDAC9–EGR1–POR	Ferroptosis-associated cell death	Sodium butyrate induced ferroptosis and restored cisplatin sensitivity through epigenetic regulation of POR
Zhang et al. [43]	CAL-27, SCC-9, CAL-27/DDP, SCC-9/DDP	Cisplatin	Acquired cisplatin resistance	miR-214-3p modulation, erastin, ferrostatin-1	miR-214-3p–GPX4	Ferroptosis-associated cell death	miR-214-3p restoration reduced GPX4, induced ferroptosis and reversed cisplatin resistance
Zhang et al. [44]	HN30, SCC-1, Cal27, HN6; HaCaT	Cisplatin	No resistant cell line reported	KIF20A silencing, erastin, RSL3, ENMD-2076	KIF20A–DHX9–SOX2–SLC7A11/GPX4	Ferroptosis-associated cell death	KIF20A promoted stemness and ferroptosis resistance; its inhibition enhanced cisplatin activity

Abbreviations: CAR, cisplatin-resistant CAL27 cells; Ce6, chlorin e6; CSC, cancer stem cell; DDP, cisplatin; DOX, doxorubicin; EGCG, epigallocatechin gallate; HNSCC, head and neck squamous cell carcinoma; HOK, human oral keratinocytes; NHOK, normal human oral keratinocytes; NOKs, normal oral keratinocytes; PDT, photodynamic therapy; PDOs, patient-derived organoids; ROS, reactive oxygen species.

**Table 2:** Main characteristics and findings of the included in vivo studies investigating ferroptosis and therapeutic resistance in HNSCC.

Study (Year)	Animal Model and Sample Size	Tumor Model	Treatment (Groups/ Intervention)	Main Target or Mechanism	Main Findings
Roh et al. [23]	Male athymic BALB/c nude mice, 6 weeks old; 10/group in the pharmacological experiment; NR for the genetic experiment	HN9-cisR cells; subcutaneous flank xenograft	SLC7A11 shRNA ± cisplatin; vehicle; cisplatin; SAS; cisplatin + SAS	xCT/SLC7A11–GSH–lipid ROS axis	SLC7A11 silencing enhanced the antitumor effect of cisplatin. SAS combined with cisplatin reduced resistant tumor growth more effectively than either treatment alone
Roh et al. [24]	Male athymic BALB/c nude mice; N NR	HN9 or HN9-cisR cells; subcutaneous flank xenograft	Artesunate; trigonelline; shNFE2L2; shKEAP1; combined treatments	KEAP1–NRF2–ARE pathway	HN9-cisR tumors were less responsive to artesunate. Pharmacological or genetic NRF2 inhibition restored ferroptosis sensitivity and enhanced tumor suppression
Zhou et al. [28]	Male athymic BALB/c nude mice, 5 weeks old; N NR	Cisplatin-resistant CAR cells; bilateral subcutaneous flank xenograft	sh-NC or sh-AEBP1 combined with cisplatin or SSZ	AEBP1–MAPK–SLC7A11/GPX4 axis	AEBP1 silencing enhanced the inhibitory effects of cisplatin and SSZ, reducing the volume and weight of cisplatin-resistant tumors
Osman et al. [31]	Male athymic nude mice; 8–12/group	HN30-P or HN30-R8 cells; orthotopic tongue xenograft	Cisplatin; NRF2 shRNA; wild-type KEAP1; IACS-6274, alone or combined with cisplatin	KEAP1/NRF2–GLS1 signaling	NRF2 activation promoted cisplatin resistance and metastatic progression. NRF2 suppression, KEAP1 restoration, or GLS1 inhibition enhanced the response of resistant tumors to cisplatin
Liu et al. [32]	Male BALB/c nude mice, 4–6 weeks old; 8/group	TSCCA/DDP cells; subcutaneous right upper-back xenograft	Vector + PBS; vector + cisplatin; Lv-TCF12 + PBS; Lv-TCF12 + cisplatin	TCF12–OTUB1–SLC7A11 axis	TCF12 overexpression inhibited resistant tumor growth and enhanced the response to cisplatin, with the combination producing stronger antitumor effects than either intervention alone
Wu et al. [34]	NOD/SCID mice; 20 animals reported for PDX generation; treatment groups shown as 5/group	Patient-derived HNSCC tumor model; subcutaneous flank implantation	Vehicle; cisplatin; iFSP1; cisplatin + iFSP1	FSP1–ACSL4 and lipid/iron metabolic regulation	FSP1 inhibition enhanced the effect of cisplatin, reduced PDX tumor growth and improved survival compared with cisplatin alone
Xu et al. [35]	BALB/c nude mice; 5/group	CAL-27 cells; subcutaneous right-flank xenograft	PBS; sorafenib; Ce6; Ce6 + laser; Sor-Ce6 NPs; Sor-Ce6 NPs + laser	Fe-mediated Fenton reaction, GSH depletion and GPX4 inhibition	Sor-Ce6 NPs combined with laser irradiation produced the greatest tumor inhibition, increased intratumoral oxidative stress and improved photodynamic activity under hypoxic conditions
Qian et al. [37]	Male BALB/c nude mice, 5 weeks old; 24 animals, 6/group	CAL27 cells; subcutaneous right-flank xenograft	Mock; cisplatin; IU1; cisplatin + IU1	USP14–FABP5–SLC7A11/GPX4 axis	Pharmacological USP14 inhibition enhanced cisplatin-mediated tumor suppression. The combination reduced tumor growth and Ki-67 expression more effectively than either treatment alone
Wang et al. [38]	Male athymic M-NSG nude mice, 5 weeks old; 12 animals, 4/group	CAL27 cells; bilateral subcutaneous flank xenograft	Scramble; cisplatin; shTPI1 + cisplatin	TPI1–SLC7A11/GPX4–TFRC axis	TPI1 silencing combined with cisplatin reduced tumor volume and weight more effectively than cisplatin alone, supporting reversal of cisplatin resistance through ferroptosis
Yu et al. [40]	C57BL/6 mice; 6/group	MOC1 cells; subcutaneous xenograft	Saline; MTX; MTX + Fer-1	ERK–KEAP1–NRF2–SLC7A11/GPX4 axis	MTX suppressed tumor growth, whereas Fer-1 attenuated its antitumor activity, indicating that ferroptosis contributed to the in vivo response
Wang et al. [42]	BALB/c nude mice; N NR	CAL27/CDDP cells; subcutaneous axillary xenograft	Cisplatin; cisplatin + NaB; cisplatin + NaB + Fer-1; additional NaB comparisons	HDAC9–EGR1–POR axis	NaB enhanced cisplatin-mediated inhibition of resistant tumor growth. Fer-1 attenuated this effect, supporting ferroptosis as a mediator of chemosensitization
Zhang et al. [43]	BALB/c nude mice; N NR	CAL-27/DDP cells; subcutaneous right-axillary xenograft	NC mimic + cisplatin; miR-214-3p mimic + cisplatin	miR-214-3p–GPX4 axis	miR-214-3p restoration enhanced cisplatin-mediated inhibition of resistant tumor growth by suppressing GPX4 and promoting ferroptosis
Zhang et al. [44]	Female BALB/c nude mice, 4 weeks old; 5/group	KIF20A-overexpressing CAL27 or KIF20A-knockout HN30 cells; subcutaneous xenograft	Erastin; Fer-1; vehicle; cisplatin; ENMD-2076; cisplatin + ENMD-2076	KIF20A–DHX9–SOX2–PI3K/AKT axis	KIF20A promoted tumor growth and ferroptosis resistance. ENMD-2076 combined with cisplatin produced greater tumor suppression than either monotherapy.

Abbreviations: ARE, antioxidant response element; CAR, cisplatin-resistant CAL27 cells; Ce6, chlorin e6; DDP, cisplatin; Fer-1, ferrostatin-1; FSP1, ferroptosis suppressor protein 1; GPX4, glutathione peroxidase 4; GSH, glutathione; HNSCC, head and neck squamous cell carcinoma; iFSP1, inhibitor of ferroptosis suppressor protein 1; MTX, methotrexate; NaB, sodium butyrate; N, number of animals; NFE2L2, nuclear factor erythroid 2-related factor 2; NPs, nanoparticles; NR, not reported; NRF2, nuclear factor erythroid 2-related factor 2; PBS, phosphate-buffered saline; PDX, patient-derived xenograft; ROS, reactive oxygen species; SAS, sulfasalazine; SLC7A11, solute carrier family 7 member 11; SSZ, sulfasalazine; TFRC, transferrin receptor; xCT, cystine/glutamate antiporter.

### 3.5. Risk of Bias and Certainty of Evidence Assessment

The certainty of the evidence from the included studies was assessed using the GRADE approach [45]. Of the 22 studies evaluated, 17 were rated as providing high-certainty evidence [23,24,25,26,28,29,31,32,34,37,38,39,40,41,42,43,44], with no serious concerns identified across the GRADE domains assessed. Five studies were rated as providing moderate-certainty evidence [27,30,33,35,36], each downgraded because of a specific methodological limitation.

Three studies [27,30,33] were downgraded for imprecision. Although these studies reported outcomes as mean ± standard deviation with corresponding statistical significance, the number of independent biological replicates was not clearly reported, limiting the assessment of the precision of the effect estimates. In addition, confidence intervals for the principal effects were not provided. The study by Xu et al. [35] was downgraded for study limitations because the cellular experiments relied exclusively on a single tumor cell line (CAL-27), without validation in an independent cell line; reliance on a single cell model without independent validation was considered a serious methodological limitation, consistent with standard GRADE practice for judging study limitations. Finally, He et al. [36] was downgraded for study limitations because no ferroptosis-specific rescue (e.g., ferrostatin-1 or liproxstatin-1) or induction experiment (e.g., erastin or RSL3) was performed to establish that the OLFM4-driven phenotype was specifically dependent on ferroptosis. Although the reported rescue experiment using N-acetylcysteine supports a role for ROS/oxidative stress, it does not establish specificity for the ferroptotic pathway (Table 3).

The methodological quality of the in vivo studies was assessed using the SYRCLE Risk of Bias tool, and the results are summarized in Table 4. Overall, most studies showed an unclear risk of bias across several methodological domains, primarily because of insufficient reporting of experimental procedures rather than explicit methodological shortcomings. Baseline characteristics, incomplete outcome data and selective outcome reporting were consistently judged as having a low risk of bias, as all studies reported the predefined outcomes and no evidence of attrition or selective reporting was identified. In contrast, sequence generation, allocation concealment, random housing, blinding of caregivers and investigators, random outcome assessment, and blinding of outcome assessment were predominantly rated as unclear risk of bias, since these procedures were rarely described in sufficient detail. Consequently, it was not possible to determine whether adequate measures had been implemented to minimize selection, performance, or detection bias. The assessment of other sources of bias varied among studies, with judgments ranging from low to high risk depending on study-specific methodological issues. Overall, the risk-of-bias assessment highlights the need for more comprehensive reporting of randomization and blinding procedures in preclinical HNSCC studies investigating ferroptosis and therapeutic resistance.

## 4. Discussion

HNSCC cells exhibit several biological characteristics that may render them susceptible to ferroptosis induction, although their sensitivity may vary due to the genetic and epigenetic heterogeneity of individual tumors [47,48]. These cells display a high dependence on iron to sustain rapid proliferative activity, resulting in increased iron uptake through transferrin receptor 1 (TfR1) and alterations in ferritin metabolism, thereby expanding the labile iron pool (LIP) and promoting oxidative stress [49]. In addition, HNSCC cells possess membranes enriched in phospholipids containing polyunsaturated fatty acids, primarily through the activity of ACSL4 and lysophosphatidylcholine acyltransferase 3, which incorporate arachidonic acid and adrenic acid into membrane phosphatidylethanolamines, a critical step in the lipid peroxidation that characterizes ferroptosis [50,51]. Despite this intrinsic susceptibility, HNSCC cells frequently undergo metabolic reprogramming accompanied by the overexpression of antioxidant defense mechanisms, including GPX4, NRF2, and FSP1, which protect against oxidative stress but also represent potential therapeutic vulnerabilities when inhibited [52,53]. Consistent with this biological background, the studies included in the present review demonstrated that ferroptosis suppression is a common adaptive mechanism associated with anticancer drug resistance in HNSCC. Although the molecular mediators differed among studies, resistant cells generally exhibited enhanced antioxidant capacity, reduced lipid peroxidation, and increased resistance to oxidative stress. These adaptations were most frequently associated with alterations in the SLC7A11–GSH–GPX4 axis and the KEAP1–NRF2 pathway, whereas mechanisms involving iron metabolism, lipid remodeling, post-transcriptional regulation, and protein stability were identified in fewer studies [23,24,25,31,32,37]. Functionally, disrupting these protective mechanisms consistently enhanced DDP sensitivity and, in several models, reduced proliferation, migration, invasion, stemness-associated traits, tumor growth, and metastatic dissemination, supporting the concept that ferroptosis resistance is an integral component of the resistant phenotype rather than a secondary consequence of treatment.

An important observation is that the current evidence is overwhelmingly centered on acquired DDP resistance. Most experimental models were generated from CAL27, SCC9, HN3, HN4, HN9, HN30, or SCC15 cells through prolonged DDP exposure, whereas studies involving cetuximab, gefitinib, methotrexate or doxorubicin were comparatively scarce. Moreover, several investigations assessed ferroptosis induction in treatment-naïve cells rather than in experimentally resistant derivatives. For example, Wang et al. [33] demonstrated that DMEFe nanoparticles potentiated doxorubicin cytotoxicity through ferroptosis-related mechanisms, but no doxorubicin-resistant model was evaluated. Similar limitations apply to studies investigating methotrexate resistance, CAY-10566, and CAV1 silencing, which primarily demonstrated treatment enhancement rather than reversal of established resistance [40]. Consequently, the available evidence is considerably stronger for platinum resistance than for other therapeutic contexts.

Among all identified mechanisms, the xCT/SLC7A11–GSH axis remains the best-supported therapeutic vulnerability. Although several upstream regulators were identified, including AEBP1 [28], circ_0000140/miR-527 [30] and TCF12–OTUB1 [32], these studies collectively converge on preservation of SLC7A11 activity as a central strategy by which resistant cells maintain GSH-dependent antioxidant defenses. Rather than representing independent pathways, these regulatory mechanisms reinforce the concept that maintenance of cystine uptake and GSH synthesis constitutes a common endpoint underlying ferroptosis resistance. A similar convergence is evident for the KEAP1–NRF2 pathway. Experimental studies demonstrated that resistant cells frequently activate NRF2-dependent antioxidant programs that limit the efficacy of ferroptosis inducers, whereas pharmacological or genetic inhibition of NRF2 restored DDP sensitivity [23,24,30]. These observations are consistent with recent reviews identifying NRF2 as one of the principal regulators of ferroptosis evasion through coordinated control of SLC7A11, GPX4, iron metabolism, and oxidative stress responses [47,48]. The antioxidant defenses mediated by NRF2 should also be considered in the context of upstream signaling pathways. In gastric cancer, inhibition of the AKT/GSK3β/NRF2 axis has been associated with ferroptosis induction and enhanced cisplatin chemosensitivity, highlighting the potential role of PI3K/AKT signaling in modulating NRF2-dependent antioxidant responses [54,55]. Similarly, in cisplatin-resistant non-small cell lung cancer, NRF2 knockdown or pharmacological inhibition enhanced cisplatin sensitivity by promoting ferroptosis, with HMOX1 identified as an important downstream mediator [56]. Together, these findings indicate that ferroptosis sensitivity is regulated by both upstream signaling networks and downstream antioxidant effectors, highlighting the importance of targeting NRF2-associated mechanisms and antioxidant adaptation to enhance the efficacy of ferroptosis-based therapies. However, whether these strategies can improve therapeutic responses in HNSCC remains to be established.

Beyond GSH metabolism, additional mechanisms broadened the regulatory landscape but currently rest on more limited evidence. Iron sequestration by FTH1 influenced intrinsic ferroptosis sensitivity across HNSCC subtypes, indicating that anatomical origin may affect therapeutic response [26]. GLS1 inhibition impaired GSH synthesis downstream of NRF2 and reduced metastatic dissemination in an orthotopic model, although validation remains limited to a single resistant cell line [31]. Similarly, OLFM4 emerged as a potential regulator of iron homeostasis and mitochondrial function after transcriptomic analysis of patient-derived organoids [36]. However, because mechanistic validation relied mainly on conventional cell lines and direct molecular interactions were not demonstrated, its precise position within the ferroptosis network remains uncertain. Several studies further suggested that ferroptosis suppression contributes not only to drug resistance but also to biological traits associated with tumor progression. FSP1 supported survival of drug-tolerant persister cells and its inhibition improved DDP efficacy in patient-derived xenografts [34], whereas USP14, TPI1, and KIF20A were associated with stemness, epithelial–mesenchymal transition, invasion, and ferroptosis resistance [37,38,44]. These observations support an emerging concept that ferroptosis resistance is closely linked to cellular plasticity and residual disease. Nevertheless, several of these studies used parental rather than resistant xenograft models, limiting conclusions regarding reversal of established resistance.

Epigenetic and post-transcriptional regulation further illustrate the complexity of ferroptosis control. The miR-214-3p/GPX4 axis [43] and the HDAC9–EGR1–POR pathway [42] both restored DDP sensitivity through enhanced lipid peroxidation. Notably, the study by Wang et al. [42] provided particularly robust mechanistic evidence because Fer-1 reversed both the cellular and in vivo therapeutic effects, directly linking chemosensitization to ferroptosis rather than nonspecific oxidative damage. Nanotechnology-based approaches may represent another promising direction by addressing pharmacological limitations of ferroptosis induction. Iron-containing nanoparticles enhanced chemotherapy through simultaneous ROS generation and inhibition of antioxidant defenses [33,35]. However, these studies largely evaluated treatment potentiation rather than established resistance, and important translational issues, including biodistribution, systemic toxicity, and long-term safety, remain insufficiently explored [57]. The potential clinical application of ferroptosis induction must also consider effects on normal tissues. Because ferroptosis contributes to oxidative tissue injury, indiscriminate activation may increase treatment-related toxicity, particularly in organs susceptible to iron-dependent damage. This concern has been highlighted for DDP-induced nephrotoxicity [58] and may also be relevant for oral mucosa and salivary glands during combined chemoradiotherapy. Consequently, current evidence supports the concept of selective tumor ferroptosis induction, rather than generalized activation, as the most realistic therapeutic objective [59].

Several methodological limitations should be considered when interpreting the available evidence. Ferroptosis was commonly inferred from combinations of ROS production, GSH depletion, lipid peroxidation, iron accumulation, and expression of SLC7A11 or GPX4, whereas ultrastructural confirmation and in vivo rescue experiments remained uncommon. Confidence in the animal studies was further limited by incomplete reporting of randomization, allocation concealment, housing procedures, and blinding, as identified by the SYRCLE assessment. Moreover, the predominance of two-dimensional cultures followed by subcutaneous xenografts, together with the limited use of orthotopic models, patient-derived organoids, patient-derived xenografts, and immunocompetent systems, reduces the translational strength of the current evidence. Future studies should prioritize standardized resistant models, clinically relevant preclinical systems, rigorous validation of ferroptosis dependence, and evaluation of predictive biomarkers such as SLC7A11, GPX4, NRF2, and FSP1, while simultaneously assessing antitumor efficacy and normal tissue toxicity.

## 5. Conclusions

This systematic review showed that ferroptosis is associated with therapeutic resistance in HNSCC, particularly in DDP-resistant models. Most of the included studies identified dysregulation in pathways involved in redox homeostasis and lipid peroxidation, especially those centered on SLC7A11/xCT, GSH, GPX4, NRF2, and FSP1. Targeting these pathways increased susceptibility to ferroptosis and improved the response to DDP across multiple experimental models, suggesting a direct role of ferroptosis in the resistant phenotype. Collectively, the available evidence indicates that ferroptosis may represent a promising therapeutic target for overcoming anticancer drug resistance.

## Figures and Tables

**Table 3:** Quality assessment of intervention-related evidence.

Study (Year)	Study Design	Study Limitation	Inconsistency	Indirectness	Imprecision	Publication Bias	Moderate/Large Effect Size	Dose Effect	Overall Quality
Roh et al. [23]	With Comparable Baseline	✓	✓	✓	✓	Unclear	✓	✓	++++
Roh et al. [24]	With Comparable Baseline	✓	✓	✓	✓	Unclear	✓	✓	++++
Han et al. [25]	With Comparable Baseline	✓	✓	✓	✓	Unclear	✓	✓	++++
Liu et al. [26]	With Comparable Baseline	✓	✓	✓	✓	Unclear	✓	✓	++++
Wang et al. [27]	With Comparable Baseline	✓	✓	✓	X: Mean ± SD and significance reported, but replicate numbers and CIs for key effects are not clearly stated.	Unclear	✓	✓	+++
Zhou et al. [28]	With Comparable Baseline	✓	✓	✓	✓	Unclear	✓	✓	++++
Jehl et al. [29]	With Comparable Baseline	✓	✓	✓	✓	Unclear	✓	✓	++++
Ma et al. [30]	With Comparable Baseline	✓	✓	✓	Mean ± SD and significance reported, but replicate numbers and CIs for key effects are not clearly stated.	Unclear	✓	✓	+++
Osman et al. [31]	With Comparable Baseline	✓	✓	✓	✓	Unclear	✓	✓	++++
Liu et al. [32]	With Comparable Baseline	✓	✓	✓	✓	Unclear	✓	✓	++++
Wang et al. [33]	With Comparable Baseline	✓	✓	✓	X: Biological replicates not clearly reported for functional experiments	Unclear	✓	✓	+++
Wu et al. [34]	With Comparable Baseline	✓	✓	✓	✓	Unclear	✓	✓	++++
Xu et al. [35]	With Comparable Baseline	X: Only one tumor cell line, CAL-27, used throughout the cellular component. This mirrors the limitation in the Pavan reference article that justified a quality downgrade when evidence relied on a single cell model.	✓	✓	✓	Unclear	✓	✓	+++
He et al. [36]	With Comparable Baseline	✓	X: No ferroptosis-specific rescue with Ferrostatin-1/Liproxstatin-1 or induction with Erastin/RSL3 to confirm OLFM4-driven phenotype depends on ferroptosis. NAC rescue shown, but this confirms ROS/oxidative stress dependence, not ferroptosis specificity.	✓	✓	Unclear	✓	✓	+++
Qian et al. [37]	With Comparable Baseline	✓	✓	✓	✓	Unclear	✓	✓	++++
Wang et al. [38]	With Comparable Baseline	✓	✓	✓	✓	Unclear	✓	✓	++++
Wang et al. [39]	With Comparable Baseline	✓	✓	✓	✓	Unclear	✓	✓	++++
Yu et al. [40]	With Comparable Baseline	✓	✓	✓	✓	Unclear	✓	✓	++++
Zhang et al. [41]	With Comparable Baseline	✓	✓	✓	✓	Unclear	✓	✓	++++
Wang et al. [42]	With Comparable Baseline	✓	✓	✓	✓	Unclear	✓	✓	++++
Zhang et al. [43]	With Comparable Baseline	✓	✓	✓	✓	Unclear	✓	✓	++++
Zhang et al. [44]	With Comparable Baseline	✓	✓	✓	✓	Unclear	✓	✓	++++

GRADE Criteria: ✓ indicates no major concerns identified; X denotes a significant limitation (or, for the moderate/large effect size and dose-response criteria, indicates that the effect was absent); Unclear signifies that the available data were insufficient to make a judgment on that item. Overall Certainty of Evidence: +++, moderate; ++++, high.

**Table 4:** Risk of bias assessment of the included in vivo studies according to the SYRCLE Risk of Bias tool.

Study	Sequence Generation	Baseline Characteristics	Allocation Concealment	Random Housing	Blinding of Caregivers and Investigators	Random Outcome Assessment	Blinding of Outcome Assessment	Incomplete Outcome Data	Selective Outcome Reporting	Other Sources of Bias	Overall Risk of Bias
Roh et al. [23]	Unclear	Low	Unclear	Unclear	Unclear	Unclear	Unclear	Low	Low	Unclear	Low
Roh et al. [24]	Unclear	Low	Unclear	Unclear	Unclear	Unclear	Unclear	Low	Low	High	High
Zhou et al. [28]	Unclear	Low	Unclear	Unclear	Unclear	Unclear	Unclear	Low	Low	High	High
Osman et al. [31]	Low	Low	Unclear	Unclear	Unclear	Unclear	Unclear	Low	Low	Low	Low
Liu et al. [32]	Unclear	Low	Unclear	Unclear	Unclear	Unclear	Unclear	Low	Low	Unclear	Low
Wu et al. [34]	Unclear	High	Unclear	Unclear	Unclear	Unclear	Unclear	Low	Low	High	High
Xu et al. [35]	Low	Low	Unclear	Unclear	Unclear	Unclear	Unclear	Low	Low	Unclear	Low
Qian et al. [37]	Low	Low	Unclear	Unclear	Unclear	Unclear	Unclear	Low	Low	Unclear	Low
Wang et al. [38]	Unclear	Low	Unclear	Unclear	Unclear	Unclear	Unclear	Low	Low	Low	High
Yu et al. [40]	Low	Low	Unclear	Unclear	Unclear	Unclear	Unclear	Low	Low	High	High
Wang et al. [42]	Unclear	Low	Unclear	Unclear	Unclear	Unclear	Unclear	Low	Low	High	High
Zhang et al. [43]	Unclear	Low	Unclear	Unclear	Unclear	Unclear	Unclear	Low	Low	Low	High
Zhang et al. [44]	Low	Low	Unclear	Unclear	Unclear	Unclear	Unclear	Low	Low	Unclear	Low

## Data Availability

The original contributions presented in this study are included in the article/Supplementary Material. Further inquiries can be directed to the corresponding author.

## Supplementary Materials

The following supporting information can be downloaded at: https://www.mdpi.com/article/10.3390/ijms27219622/s1.
