# Supplementary material for: Role of Ferroptosis in Anticancer Drug Resistance in Head and Neck Squamous Cell Carcinoma: A Systematic Review of Preclinical Studies
Source: Int J Mol Sci. 2026 Oct 27;27(21):9622. doi: 10.3390/ijms27219622 (PMC13762916; doi:10.3390/ijms27219622)
Supplement: Supplementary file 1 [file ijms-27-09622-s001.zip › ijms-4654494-supplementary.pdf]

International Journal of
*Molecular Sciences*

MDPI

**Supplementary Table S1.** Search strategies with appropriate key words and MeSH terms.

| Database | Strategy |
| --- | --- |
| PubMed/Medline | ("Ferroptosis"[Mesh] OR ferroptosis OR "iron-dependent cell death") AND ("Carcinoma, Squamous Cell"[Mesh] OR "oral squamous cell carcinoma" OR OSCC OR "oral cancer" OR "oral carcinoma")<br>("Ferroptosis"[Mesh] OR ferroptosis OR "iron-dependent cell death") AND ("Carcinoma, Squamous Cell"[Mesh] OR "oral squamous cell carcinoma" OR OSCC OR "oral cancer" OR "oral carcinoma") AND (in vitro OR in vivo OR preclinical OR "cell line*" OR "animal model*" OR mouse OR mice OR rat*) |
| Science Direct | (ferroptosis OR "iron-dependent cell death") AND ("oral squamous cell carcinoma" OR OSCC OR "oral cancer")<br>(ferroptosis OR "iron-dependent cell death") AND ("oral squamous cell carcinoma" OR OSCC OR "oral cancer") AND (preclinical OR "in vitro" OR "in vivo" OR "cell line" OR "animal model") |
| Scopus | TITLE-ABS-KEY (ferroptosis OR "iron dependent cell death") AND TITLE-ABS-KEY ("oral squamous cell carcinoma" OR OSCC OR "oral cancer" OR "oral carcinoma")<br>TITLE-ABS-KEY (ferroptosis OR "iron dependent cell death") AND TITLE-ABS-KEY ("oral squamous cell carcinoma" OR OSCC OR "oral cancer" OR "oral carcinoma") AND TITLE-ABS-KEY (in vitro OR in vivo OR preclinical OR "cell line*" OR "animal model*" OR mouse OR mice OR rat*) |
| EMBASE | ('ferroptosis'/exp OR ferroptosis OR 'iron dependent cell death') AND ('oral squamous cell carcinoma'/exp OR 'oral cancer'/exp OR OSCC)<br>('ferroptosis'/exp OR ferroptosis OR 'iron dependent cell death') AND ('oral squamous cell carcinoma'/exp OR 'oral cancer'/exp OR OSCC) AND (in vitro OR in vivo OR preclinical OR 'cell line'/exp OR 'animal model'/exp OR mouse OR rat) |

**Supplementary Table S2.** List of excluded studies along with reasons for exclusion (n=11).

| Author (Year) | Title and DOI | Reason for Exclusion |
| --- | --- | --- |
| Cheng et al. (2023) | Mechanism of ferroptosis in cisplatin killing tongue squamous cell carcinoma. DOI: 10.16073/j.cnki.cjcpt.2023.08.04 | Full text not available |
| Fan et al. (2025) | LMO4 promotes OSCC progression by inducing RAB17 degradation and ferroptosis resistance. DOI: 10.1038/s41419-025-08171-1 | Study not focused on resistance |

| Author (Year) | Title and DOI | Reason for Exclusion |
|---|---|---|
| Gupta et al. (2025) | DNA Damage-Induced Ferroptosis: A Boolean Model Regulating p53 and Non-Coding RNAs in Drug Resistance. DOI: 10.3390/proteomes13010006 | Study based on in silico analyses |
| Shanmugam (2024) | Exploring ferroptosis induction: A promising approach to enhance OSCC treatment efficacy and overcome drug resistance. DOI: 10.1158/0008-5472.CAN-23-4042 | Full text not available |
| Li et al. (2022) | Interleukin-6 facilitates tumor progression by inducing ferroptosis resistance in head and neck squamous cell carcinoma. DOI: 10.1016/j.canlet.2021.12.011 | Study not focused on resistance |
| Liu et al. (2025) | Metal-dependent cell death resistance contribute to lymph node metastasis of oral squamous cell carcinoma. DOI: 10.3389/fcell.2025.1541582 | Study based on in silico analyses |
| Takami et al. (2025) | Amoxicillin, a β-lactam antibiotic, enhances cisplatin sensitivity in cancer cells affecting mitochondria. DOI: 10.1016/j.bbrc.2025.151888 | Lack of focus on ferroptosis |
| Wang et al. (2025) | Identification of a novel signature derived from ferroptosis-related genes to predict prognosis, immune landscape and chemotherapeutic sensitivity in head and neck squamous cell carcinoma. DOI: 10.1016/j.jormas.2025.102392 | Study based on in silico analyses |
| Wang et al. (2025) | Leveraging programmed cell death patterns to predict prognosis and therapeutic sensitivity in OSCC. DOI: 10.1111/odi.15139 | Lack of focus on ferroptosis |
| Yan et al. (2025) | The splicing factor SRSF6 mediates ferroptosis resistance in head and neck squamous cell carcinoma through induction of stearoyl-CoA | Study not focused on resistance |

| Author (Year) | Title and DOI | Reason for Exclusion |
|---|---|---|
| | desaturase. DOI: 10.1016/j.jbc.2025.110509 | |
| Zhu et al. (2026) | VAMP7 governs ferroptosis suppression and cisplatin resistance in esophageal cancer: a dual-targeting therapeutic paradigm. DOI: 10.1186/s12935-025-04153-7 | Study did not explore HNSCC |

https://doi.org/10.3390/ijms27219622
